# Subtypes of Persistent Postural-Perceptual Dizziness

**DOI:** 10.3389/fneur.2021.652366

**Published:** 2021-04-16

**Authors:** Chihiro Yagi, Yuka Morita, Meiko Kitazawa, Tatsuya Yamagishi, Shinsuke Ohshima, Shuji Izumi, Kuniyuki Takahashi, Arata Horii

**Affiliations:** Department of Otolaryngology Head and Neck Surgery, Niigata University Graduate School of Medical and Dental Sciences, Niigata, Japan

**Keywords:** chronic dizziness, persistent postural-perceptual dizziness, subtypes, factor analysis, cluster analysis

## Abstract

**Background:** Persistent postural-perceptual dizziness (PPPD) is a persistent chronic vestibular syndrome exacerbated by upright posture/walking, active or passive motion, and exposure to moving or complex visual stimuli. PPPD has four precursors: phobic postural vertigo, space-motion discomfort, visual vertigo, and chronic subjective dizziness. These four diseases share clinical features that form the basis of the diagnostic criteria for PPPD. Semiological similarities do not necessarily mean that PPPD is a single entity. However, if PPPD is not a single disorder but just a composite of four precursors, it may be subdivided according to the characteristics of each precursor.

**Objective:** To test whether PPPD is a single disorder, we attempted a subtyping of PPPD.

**Methods:** One-hundred-eight untreated patients with PPPD were enrolled in the study, who filled out the Niigata PPPD Questionnaire (NPQ) that consists of 12 questions on exacerbating factors for PPPD. A factor analysis of the patients' answers to the NPQ and a subsequent cluster analysis of the patients with PPPD using factors revealed by the factor analysis were performed. To validate our cluster classification, cluster differences were assessed using analysis of variance. Multiple comparison analyses were performed on demographical data, precipitating diseases, the Dizziness Handicap Inventory, the Hospital Anxiety and Depression Scale, and several vestibular tests to characterize each cluster.

**Results:** Factor analysis revealed three underlying factors among the exacerbating factors in the NPQ. Exacerbation by visual stimuli (visual factor) accounted for 47.4% of total variance in the questionnaire. Exacerbation by walking/active motion (active-motion factor) and by passive motion/standing (passive-motion/standing factor) accounted for 12.0 and 7.67% of variance, respectively. Cluster analysis revealed three clusters: the visual-dominant subtype (*n* = 49); the active motion-dominant subtype (*n* = 20); and the mixed subtype (*n* = 39). The patients in the active motion-dominant subtype were significantly older than those in the visual-dominant subtype. There were no significant differences among the subtypes in other demographical data or conventional vestibular tests.

**Conclusions:** The most common main exacerbating factor of PPPD was the visual factor. PPPD may be categorized into three subtypes. Conventional vestibular tests failed to point the characteristics of each subtype.

## Introduction

Persistent postural-perceptual dizziness (PPPD), which has been included in the 11th revision of the International Classification of Diseases, is a persistent chronic vestibular syndrome typically preceded by acute vestibular disorders (1). The core vestibular symptoms of PPPD are dizziness, unsteadiness, and/or non-spinning vertigo that are exacerbated by an upright posture (standing or walking), active or passive motion, and exposure to moving visual stimuli or complex visual patterns (1). PPPD presents with chronic vestibular symptoms. Nonetheless, it is not a disorder of the vestibular periphery; rather, it is considered a functional disorder caused by shifts in the functioning of spatial orientation systems to favor visual or somatosensory/proprioceptive stimuli over vestibular inputs (2).

PPPD has four precursors: phobic postural vertigo (PPV), space-motion discomfort (SMD), visual vertigo (VV), and chronic subjective dizziness (CSD) (3–7). These four diseases share clinical features that form the basis of the diagnostic criteria of PPPD (1). Exacerbation by upright posture, active or passive motion, complex visual pattern, and motion of self or objects in the environment have been emphasized in PPV, SMD, VV, and CSD, respectively (1). Semiological similarities do not necessarily indicate that PPPD is a single entity. However, if PPPD is not a single disorder but just a composite of four precursors, it may be subdivided according to the characteristics of each precursor such as the postural- or visual-provocation dominant subtypes. To test whether PPPD is a single disorder, we attempted a subtyping of PPPD.

Here, we aimed to investigate the possible subtypes of PPPD, based on the exacerbating factors assessed by the symptom scale for PPPD, the Niigata PPPD Questionnaire (NPQ) (8), using factor and cluster analysis. Given that specific vestibular tests that can identify PPPD are not yet available, certain vestibular tests could potentially be associated with one specific subtype of PPPD, e.g., visual dependence of postural control in the visual-provocation dominant subtype. To characterize each subtype of PPPD, we compared the demographical data, results of vestibular tests, precipitating diseases, and psychiatric status among the different subtypes.

## Materials and Methods

### Patients

In total, 108 untreated patients with PPPD who visited the Department of Otolaryngology Head and Neck Surgery at Niigata University Medical and Dental Hospital between January 2018 and August 2020 were enrolled in the study. PPPD was defined by using the Barany Society criteria (1). There were 32 men and 76 women with a mean age of 50.6 years and standard deviation (SD) of 15.1 years (men, 53.8 years [SD, 13.5 years]; women, 49.2 years [SD 15.5 years]). There were no differences in age between the male and female groups (*t*-test, *p* = 0.149). The precipitating conditions for PPPD among patients are shown in Table 1. Since our patients were recruited from the ENT department, most patients had vestibular precipitants. Thus, our study may suffer from a patient selection bias.

**Table 1:** Precipitating conditions for patients with persistent postural-perceptual dizziness (*n*).

**Vestibular disorders**	***n* = 78**	**Non-vestibular disorders**	***n* = 19**	**No specific precipitants**	***n* = 11**
Acute attack of peripheral vestibular vertigo	*n* = 31	Chronic anxiety disorders	*n* = 12		
BPPV	*n* = 20	Post-traumatic brain injury	*n* = 3		
Meniere's disease	*n* = 15	Orthostatic dysfunction	*n* = 2		
Sudden deafness with vertigo	*n* = 5	Cerebellar infarction	*n* = 1		
Vestibular neuritis	*n* = 4	Drug-induced vertigo	*n* = 1		
Vestibular migraine	*n* = 1				
Delayed endolymphatic hydrops	*n* = 1				
Labyrinthitis due to cholesteatoma	*n* = 1				

*BPPV, Benign paroxysmal positional vertigo*.

### The Niigata PPPD Questionnaire (NPQ)

The NPQ (Table 2) evaluates the degree of symptom exacerbation by three characteristic factors based on the diagnostic criteria, namely: upright posture/walking, motion, and visual stimulation (8). Each factor is assessed by four questions, with a total of 12 questions in the questionnaire. Q3, 6, 7, and 11 pertain to the upright posture (standing or walking); Q1, 5, 9, and 12 pertain to active or passive motion; and Q2, 4, 8, and 10 pertain to visual stimulation. Each question is scored from 0 (no symptom) to 6 (unbearable); therefore, the maximum score for each factor is 24, and the maximum score for all three factors together is 72. It was demonstrated that a visual stimulation score of 9 has the best sensitivity (82%) and specificity (74%) for discriminating PPPD from control vestibular diseases (8).

**Table 2:** Niigata Persistent Postural-Perceptual Dizziness Questionnaire (8). Instructions: The purpose of this questionnaire is to identify difficulties in daily life that you may be experiencing due to dizziness. Please indicate your answer by circling the number that best describes the extent to which you have been affected during the past week. When you avoid performing these actions, you should circle the number 6.

	**None Unbearable**
Q1. Quick movements such as standing up or turning your head	0 1 2 3 4 5 6
Q2. Looking at large store displays	0 1 2 3 4 5 6
Q3. Walking at your natural pace	0 1 2 3 4 5 6
Q4. Watching TV or movies with intense movement	0 1 2 3 4 5 6
Q5. Riding a car, bus, or train	0 1 2 3 4 5 6
Q6. Sitting upright in a seat without back and arm support	0 1 2 3 4 5 6
Q7. Standing without touching fixed objects	0 1 2 3 4 5 6
Q8. Watching a scroll screen on PC or smartphone	0 1 2 3 4 5 6
Q9. Performing activities such as housework or light exercise	0 1 2 3 4 5 6
Q10. Reading small letters in a book or newspaper	0 1 2 3 4 5 6
Q11. Striding at a rapid pace	0 1 2 3 4 5 6
Q12. Riding an elevator or escalator	0 1 2 3 4 5 6

The NPQ is specifically designed to assess the symptoms of PPPD. Therefore, a separate questionnaire is needed to assess the impact of dizziness on daily life and psychiatric symptoms that may exacerbate dizziness, such as depression and anxiety. To evaluate these symptoms, we included the following two questionnaires in this study.

### Measures

#### Dizziness Handicap Inventory (DHI)

The DHI is a standard questionnaire that quantitatively evaluates the degree of handicap in the daily life of patients with vestibular disorders and comprises 25 questions (9, 10). The total score ranges from 0 (no disability) to 100 (severe disability).

#### Hospital Anxiety and Depression Scale (HADS)

The HADS is a self-reported questionnaire comprising anxiety and depression subscales. Each HADS subscale is assessed using seven questions (11). The response to each question is scored from 0 (not at all) to 3 (most of the time, very often); therefore, the total score for each HADS subscale is 21, and the full HADS score is 42.

### Vestibular Tests

#### Posturography

The patients underwent static posturography on a solid or rubber foam surface using Gravicoda® (ANIMA Corp., Japan), with their eyes open and closed. The foam ratio (posturography with/without foam) with eyes closed was used as an indicator for somatosensory dependence of postural control, whereas the Romberg ratio on foam was used as an indicator for visual dependence (12).

#### Bithermal Caloric Testing (BCT)

The BCT was carried out using air at 26°C and at 45°C each for 60 s. Each external auditory canal was stimulated separately with a 5-min interval between the stimulations. Maximum slow-phase velocity was measured using electronystagmography and canal paresis % (CP%) was calculated using the Jongkee's index formula (13).

#### Video Head Impulse Test (vHIT)

The horizontal vestibulo-ocular reflex (VOR) was evaluated by vHIT (Eye See Cam VOG®, Zero C Seven, Inc., Japan). Small-amplitude, high-acceleration, and passive head rotations were applied around the horizontal plane (yaw) at ~20° with a mean velocity of 150°/s, mean acceleration of 1000–2500°/s^2^, and the patients' gaze fixed at a target placed 1.5 m in front of him/her. At least 7 suitable head rotational impulses to the right and left were recorded during each test.

#### Cervical- and Ocular Vestibular-Evoked Myogenic Potentials (cVEMP and oVEMP)

cVEMP and oVEMP were used to assess the saccular and utricular function, respectively, using the Neuropack® system (Nihon Koden, Japan). The click (0.1-ms rarefactive square waves of 105-dB nHL) was used to induce cVEMP. For the recording of oVEMP, a hand-held electromechanical vibrator (Minishaker®, Bruel & Kjaer, Denmark) fitted with a short bolt terminating in a plastic cap was used. The vibrator delivered a 500-Hz tone burst (4-ms plateau and 1-ms rise and fall) on the subject's skull at the Fz (midline of the hairline). Amplitudes and latencies were measured at the response peaks, which occurred at ~13 and 23 ms for the cVEMP and 10 and 15 ms for the oVEMP, depending on the stimulus. The difference between the peak amplitudes was used to give the peak-to-peak (PP) amplitude. To compare the two ears, the asymmetry ratio (AR) was calculated using the formula (right - left) / (right + left) x 100 (%) on the raw PP amplitude (14).

### Statistical Analysis

Our statistical analysis followed a three-step procedure. In the first step, exploratory factor analysis with promax rotation was used to study the factorial structure of symptoms according in the NPQ scores, which can identify latent common factors associated with the 12 questions of the NPQ. When selecting the number of factors, only factors with eigenvalues of 1.00 or higher were selected. The factor scores, which were to be used in the subsequent cluster analysis, were calculated for all patients by using regression analysis. All factor scores were transformed to mea *n* = 0 and SD = 1.

The second step was a cluster analysis of the factors observed in the previous step to identify groups of objects that were similar to each other but different from objects in other groups. The clustering was performed based on a hierarchical method that considered the Ward clustering method based on the Euclidean distance and may be visualized in a dendrogram. To validate the cluster classification, cluster differences were assessed using the variance analysis by Tukey method.

Finally, the clusters observed in the previous step were characterized. A chi-square test was performed on sex differences, and Fisher's exact probability test was performed on precipitating diseases. A Kruskal–Wallis test followed by *post-hoc* Dann–Bonferroni test was performed on age, disease duration, DHI, HADS, and several vestibular tests to characterize the background of each cluster.

All statistical analyses were performed using SPSS version 26.0 for Windows. A *p*-value < 0.05 was considered statistically significant.

## Results

### Step 1: Factor Analysis

When we first conducted a factor analysis using the 12 questions, Q6 showed a commonality estimate <1 and was observed to be an inappropriate variable for the analysis; therefore, Q6 was excluded and the factor analysis was conducted using 11 questions. The factor analysis yielded three factors underlying the associations among the 11 symptoms in the NPQ (Table 3). Factor 1, Factor 2, and Factor 3 accounted for 47.4, 12.0, and 7.67% of the total variance in the questionnaire, respectively. Remaining ~33% of the variance could be attributed to other minor factors with eigenvalues <1.00. As Factor 1 was associated with the questions pertaining to visual stimuli, we regarded it as the “visual factor.” As Factor 2 was associated with the questions pertaining to walking and active motion, we regarded it as the “active-motion factor.” Finally, as Factor 3 was associated with the questions pertaining to passive motion and standing, we regarded it as the “passive-motion/standing factor.” The factor scores obtained by factor analysis were fed into the subsequent cluster analysis.

**Table 3:** The factor analysis performed on the Niigata PPPD Questionnaire (NPQ) scores.

**Questions of the NPQ**	**Factor 1**	**Factor 2**	**Factor 3**
	**Visual factor**	**Active-motion factor**	**Passive-motion/**
			**standing factor**
Q8. Watching a scroll screen on PC or smartphone	0.900**	−0.060	−0.034
Q4. Watching TV or movies with intense movement	0.833**	−0.003	0.004
Q1. Quick movements such as standing up or turning your head	0.409**	0.299	0.049
Q10. Reading small letters in a book or newspaper	0.401**	0.136	0.206
Q2. Looking at large store displays	0.348*	0.227	0.130
Q3. Walking at your natural pace	0.030	0.928**	−0.201
Q11. Striding at a rapid pace	−0.126	0.845**	0.221
Q9. Performing activities such as housework or light exercise	0.124	0.551**	0.074
Q12. Riding an elevator or escalator	−0.045	0.066	0.809**
Q5. Riding a car, bus, or train	0.261	−0.216	0.642**
Q7. Standing without touching fixed objects	−0.003	0.137	0.518**

Q6 was excluded because it showed a commonality estimate >1 and was judged to be an inappropriate variable for analysis. The single asterisk (*) indicate loadings > 0.2 and the double asterisks (**) indicate loadings > 0.4. The bigger loading is considered priorities for factor interpretation.

### Step 2: Cluster Analysis

**Figure 1:**
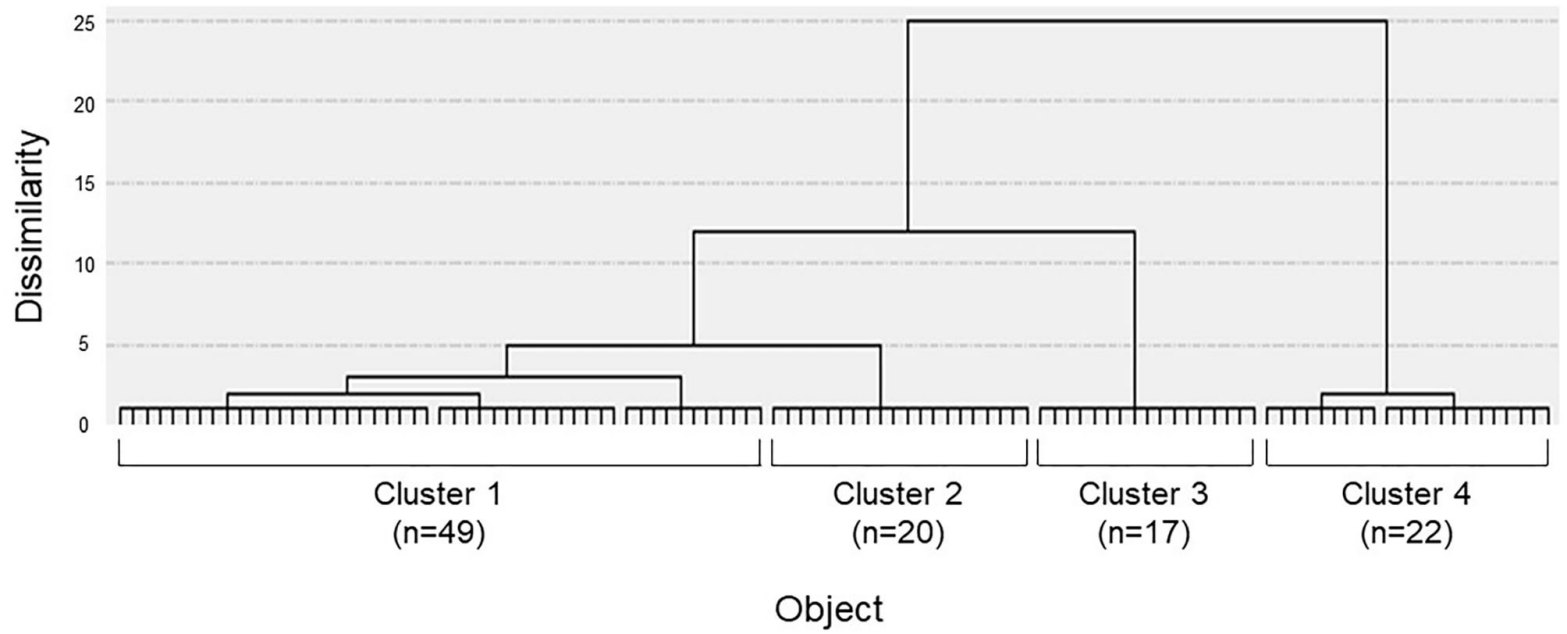
Dendrogram of patients constructed by the Ward clustering method. Four clusters were initially selected by using the factor scores obtained through the previous factor analysis.

**Figure 2:**
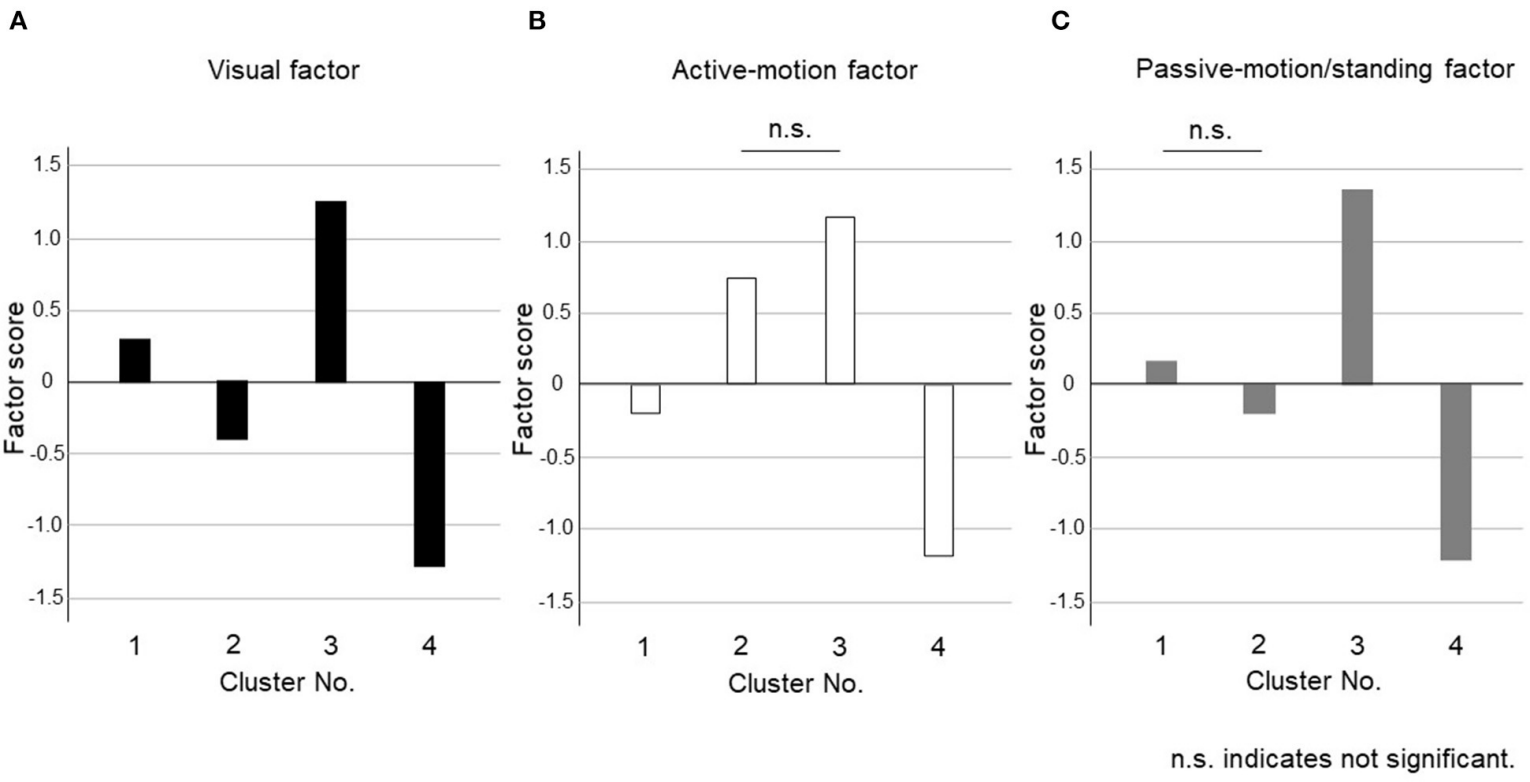
Cluster differences assessed using the variance analysis by Tukey method for four clusters. The factor scores were shown in each cluster for **(A)** Visual factor, **(B)** Active-motion factor, and **(C)** Passive-motion/standing factor. The third (severe) and the fourth (mild) clusters, which were characterized by the symptom severity but not by the exacerbating factors, were combined into one cluster, which was renamed cluster 3'.

**Figure 3:**
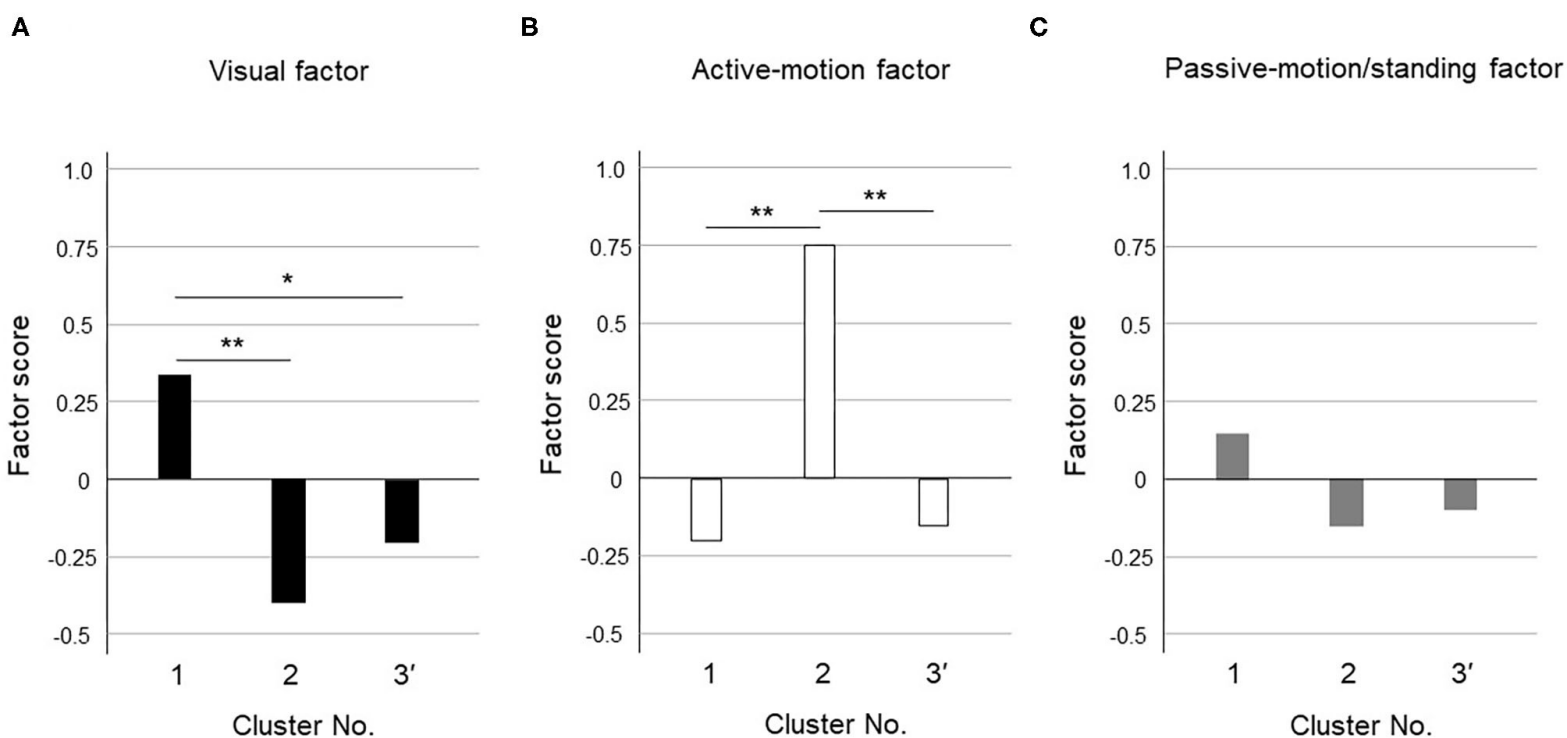
Cluster differences assessed using the variance analysis by Tukey method for three clusters. The factor scores were shown in each cluster for **(A)** Visual factor, **(B)** Active-motion factor, and **(C)** Passive-motion/standing factor. The cluster 1 had a high visual factor score, so we termed it the “visual-dominant subtype.” The cluster 2 had a high active-motion factor score, so we termed it the “active motion-dominant subtype.” The cluster 3' had no dominant exacerbation factors but was seemed to be exacerbated equally by all three factors, which was termed the “mixed subtype”. The single asterisk (*) indicate *p* < 0.05 and the double asterisks (**) indicate *p* < 0.01 by Tukey method.

The dendrogram from the cluster analysis is presented in Figure 1. In the absence of an objective method to optimally select the number of clusters, we initially selected 4 clusters for discriminating the features and labeling easily. Cluster differences assessed using the variance analysis by Tukey method are presented in Figure 2. The first cluster had a high visual factor score, and the second cluster had a high active-motion factor score. The third and fourth clusters had high and low scores for all three factors, respectively. In other words, both of the third and the fourth clusters in Figure 2 had no dominant exacerbations by visual-, active-motion, nor passive motion/standing factors. Therefore, the third (severe) and the fourth (mild) clusters, which were characterized by the symptom severity but not by the exacerbating factors, were combined into one cluster, which was renamed cluster 3'. Thus, PPPD patients were finally clustered into 3 subtypes based on their exacerbation factors (Figure 3).

Figure 3 shows the results of the variance analysis by Tukey method to discriminate the features among the 3 clusters. Forty-nine patients belonged to cluster 1, 20 to cluster 2, and 39 to cluster 3', respectively. The cluster 1 had a high visual factor score, so we termed it the “visual-dominant subtype.” The cluster 2 had a high active-motion factor score, so we termed it the “active motion-dominant subtype.” The cluster 3' had no dominant exacerbation factors but was seemed to be exacerbated equally by all three factors, which was termed the “mixed subtype.”

### Step 3: Characteristics of Each Subtype

**Figure 4:**
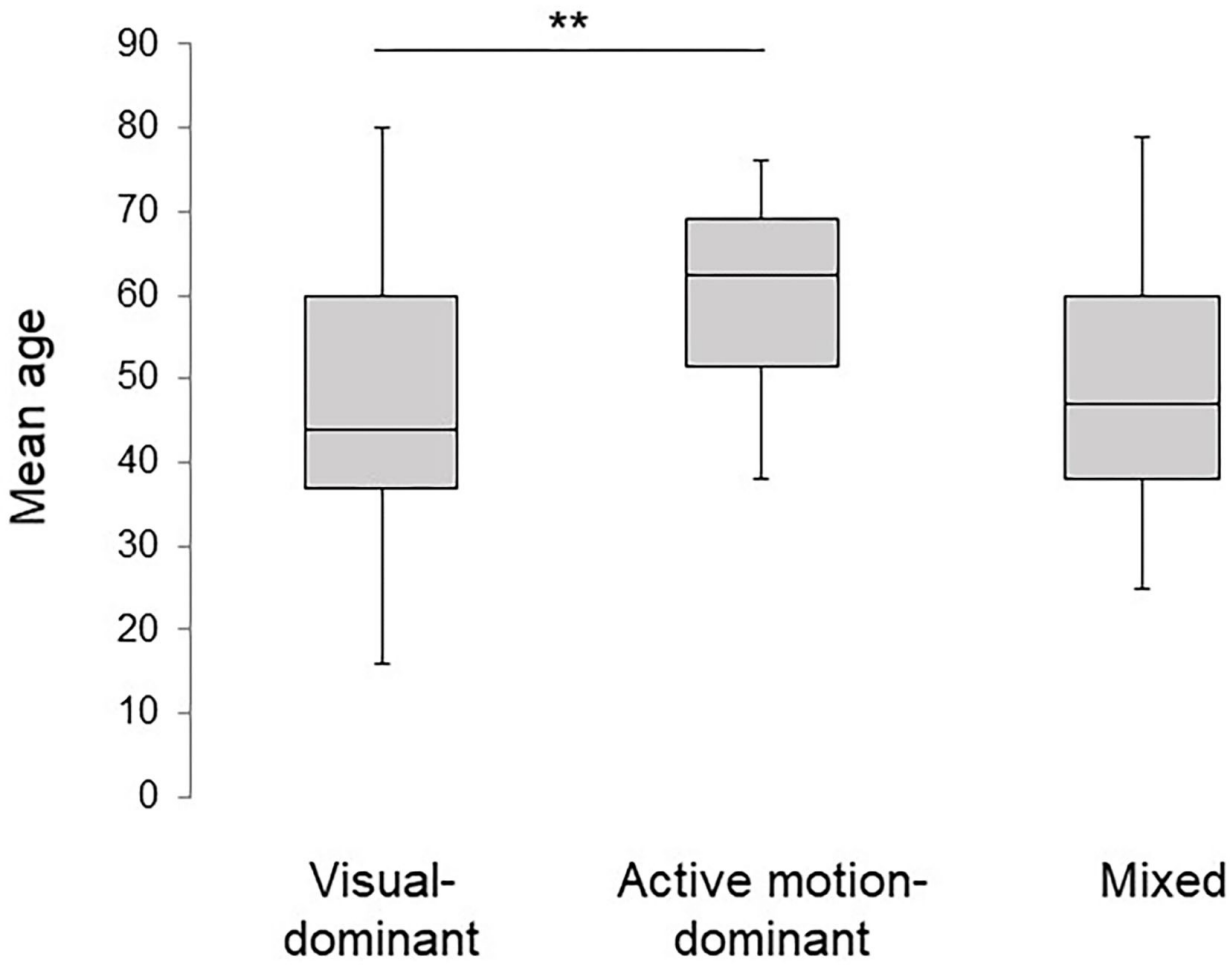
Comparisons of age between subtypes. The mean age of the active motion-dominant subtype was significantly older than that of the visual-dominant subtype. The double asterisks (**) indicate *p* < 0.01 by Dann-Bonferroni method.

The basic demographic characteristics of the three subtypes are shown in Table 4. The mean age in the active motion-dominant subtype was significantly older than that in the visual-dominant subtype (bold in Table 4, Figure 4). No significant differences were observed among the subtypes regarding the sex, disease duration and precipitating conditions. Table 5 shows the results of the Kruskal–Wallis method on DHI, HADS, and several vestibular tests. No significant differences were observed among the three subtypes regarding the DHI scores, HADS scores and the results of vestibular tests.

**Table 4:** Basic demographic characteristics of the three subtypes.

**Variables**	**All**	**Visual-dominant (Cluster 1)**	**Active motion-dominant (Cluster 2)**	**Mixed (Cluster 3')**	***p*-value**
Sample size [Male/Female]	108 [32/76]	49 [11/38]	20 [6/14]	39 [15/24]	0.270
Age, years (Mean ± SD)	50.6 ± 15.1	47.7 ± 15.4	59.9 ± 12.4	49.5 ± 14.6	<**0.01**
Disease duration, month (Mean ± SD)	28.5 ± 40.2	26.7 ± 31.3	23.1 ± 37.2	33.6 ± 51.2	0.367
Precipitating conditions, n					
Vestibular disorders	78	37	13	28	0.056
Non-vestibular disorders	19	11	4	4	
No specific precipitants	11	1	3	7	

*Bold indicates a significant difference in the Kruskal-Wallis test*.

**Table 5:** Clinical characteristics of the three subtypes.

**Variables**	**All (*n* = 108)**	**Visual-dominant (*n* = 49)**	**Active motion-dominant (*n* = 20)**	**Mixed (*n* = 39)**	**Kruskal–Wallis *p*-value**
	**Mean ± SD**	**Mean ± SD**	**Mean ± SD**	**Mean ± SD**	
DHI (total score)	51.3 ± 20.8	50.2 ± 18.9	55.7 ± 19.8	50.3 ± 23.9	0.471
HADS (total score)	16.7 ± 7.24	17.2 ± 7.40	15.6 ± 8.25	16.7 ± 6.69	0.610
Foam ratio	2.10 ± 0.62 (*n* = 100)	2.16 ± 0.63 (*n* = 45)	2.12 ± 0.69 (*n* = 18)	2.00 ± 0.60 (*n* = 37)	0.410
Romberg ratio	1.89 ± 0.62 (*n* = 100)	1.90 ± 0.70 (*n* = 45)	1.78 ± 0.38 (*n* = 18)	1.94 ± 0.63 (*n* = 37)	0.782
CP, %	20.7 ± 22.3 (*n* = 96)	21.1 ± 22.9 (*n* = 41)	17.9 ± 17.5 (*n* = 18)	21.7 ± 24.3 (*n* = 37)	0.910
vHIT gain (rt.)	0.97 ± 0.21 (*n* = 46)	1.02 ± 0.15 (*n* = 25)	0.83 ± 0.29 (*n* = 7)	0.93 ± 0.24 (*n* = 14)	0.325
vHIT gain (lt.)	1.01 ± 0.15 (*n* = 46)	1.01 ± 0.07 (*n* = 25)	0.98 ± 0.10 (*n* = 7)	1.03 ± 0.25 (*n* = 14)	0.571
cVEMP (asymmetry ratio), %	26.6 ± 28.9 (*n* = 78)	21.6 ± 23.6 (*n* = 34)	33.8 ± 38.3 (*n* = 13)	29.1 ± 30.3 (*n* = 31)	0.682
oVEMP (asymmetry ratio), %	21.4 ± 26.6 (*n* = 66)	17.8 ± 18.3 (*n* = 29)	32.0 ± 40.0 (*n* = 9)	21.7 ± 29.4 (*n* = 28)	0.752

*DHI, Dizziness Handicap Inventory; HADS, Hospital Anxiety and Depression Scale; CP, canal paresis; vHIT, Video head impulse test; cVEMP and oVEMP, Cervical- and ocular vestibular-evoked myogenic potentials*.

## Discussion

The questions in the NPQ (Table 2) reflect the three exacerbating factors of PPPD described in the diagnostic criteria (1), namely: upright posture (standing or walking), passive or active motion, and visual stimulation. However, the results of our factor analysis (Table 3) demonstrated that walking and standing inducement, both of which belong to the upright posture factor in the diagnostic criteria of PPPD, were allocated to different factors: the former to Factor 2, and the latter to Factor 3. Similarly, active motion was allocated to Factor 2 and passive motion to Factor 3. Therefore, the exacerbating factors described in the diagnostic criteria of PPPD were rearranged into Factors 1–3, based on the factor analysis. Factor 1 showed high loading for exacerbation by visual stimulation; therefore, it was termed the visual factor. Factor 2 had high loadings for exacerbation by walking and active motion; therefore, it was termed the active-motion factor. Furthermore, factor 3 had high loadings for exacerbation by passive motion and standing; therefore, it was termed the passive-motion/standing factor. As such, the factor analysis demonstrated the rearrangement of exacerbating factors (Factor 1, visual factor; Factor 2, active-motion factor; and Factor 3, passive-motion/standing factor), compared with the original description of the diagnostic criteria of PPPD (upright posture (standing or walking), passive or active motion, and visual stimulation).

Factor 1, Factor 2, and Factor 3 accounted for 47.4, 12.0, and 7.67% of the total variance in the questionnaire, respectively. This finding suggested that exacerbation by the visual factor was the most common core characteristic in patients with PPPD, followed by exacerbation by active-motion and passive-motion/standing factors, respectively.

Regarding the mechanisms of exacerbation by Factor 1 (visual factor) or Factor 2 (active-motion factor), increased visual or somatosensory dependence of spatial orientation, respectively, may be a possible explanation (15, 16). Nonetheless, our results on the Romberg ratio (1.89 ± 0.62) and the foam ratio (2.10 ± 0.62) did not show any abnormalities in visual/somatosensory dependence of postural control (Table 5), probably because our simple experimental method was not sensitive enough to detect differences in postural control dependency. Exacerbation by Factor 3 (passive-motion/standing factor) might have been due to a lower threshold for engaging closed-feedback mechanisms from open-loop regulations to adjust posture when patients with PPPD are moving passively in vehicles, or when they are standing upright, which may be related to the patients' psychiatric status (17). While evidence is still needed to demonstrate all these mechanisms, given that patients with PPPD do not show any apparent abnormalities in the vestibular periphery, PPPD is assumed to be a functional disease of the central nervous system (1).

Our cluster analysis identified three subtypes of PPPD. As long as they were diagnosed with PPPD, the subjects should be more or less exacerbated by all factors. Among them, the visual factor score was significantly higher in cluster 1 and the active-motion factor score was significantly higher in cluster 2 (Figure 3); therefore, cluster 1 and cluster 2 were designated as a visual-dominant subtype and an active motion-dominant subtype, respectively. Cluster 3' was a group of subjects with uniformly high or low factor scores for all exacerbating factors, and was considered to be a cluster that responded equally to multiple exacerbating factors; therefore, cluster 3' was designated as a mixed subtype. Each cluster may have a different degree of shift toward visual or somatosensory dependence. In contrast to visual and active-motion factors (Figures 3A,B), passive motion/standing factors did not largely differ among the three clusters (Figure 3C). Overall distribution of passive motion/standing factors to three clusters seemed to be resembling visual factors rather than active-motion factor, e.g., highly distributed to the cluster 1 and lowly to clusters 2 and 3', suggesting that passive motion/standing factors may be recognized as perceptual factors in conjunction with the visual factor.

In the comparison of the demographical characteristics by subtype, the participants with the active motion-dominant subtype were significantly older than those with the visual-dominant subtype (Figure 4). It has previously been reported that the older the patient is, the more visually dependent he or she tends to be in postural control (18). As the older patients were already visually dependent, they may have responded to the precipitating vertiginous conditions by becoming more somatosensory dependent. This could account for the younger distribution of the visual-dominant subtype than active motion-dominant subtype.

Comparisons of vestibular function tests demonstrated no differences in visual/somatosensory dependence of posture (Romberg ratio/foam ratio), horizontal canal function (CP% and vHIT), and otolith function (cVEMP and oVEMP) among the subtypes, suggesting that the differences in subtype characteristics were not derived from the type and severity of vestibular dysfunction (Table 5). Since 30 of 108 patients had no vestibular precipitants, this could partly account for no correlation between subtypes and vestibular tests. There were also no differences in sex, disease duration, precipitating conditions, DHI, and HADS among the subtypes (Tables 4, 5). Correlates with each subtype should be explored among factors other than conventional vestibular function tests, general dizziness rating scale, and psychiatric conditions. Given that the PPPD may have the central nature as functional disorders (1), functional MRI could potentially give such insights (19, 20).

Under the assumption that PPPD is not a single disease, cluster analysis should reveal four clusters characterized by the four precursors of PPPD, i.e., exacerbation by upright posture (PPV), active or passive motion (SMD), complex visual pattern (VV), and motion of self or objects in the environment (CSD). Therefore, our results, which demonstrated three subtypes (the visual-dominant subtype, the active motion-dominant subtype, and the mixed subtype) with rearranged characteristics, suggest that PPPD may be a single entity, or at least, not a composite of four different diseases.

## Limitations and Conclusions

There are several limitations to our study. Factor and cluster analyses may yield subjective results, rather than a unique answer, since they depend on the included variables and the selected number of factors or clusters. Our sample size was relatively small for a cluster analysis; thus, it was impossible to include other factors such as precipitating conditions and comorbid diseases into the cluster analysis. The characteristics of each subtype could not be determined based on the vestibular tests and were only proven through subjective symptoms.

In conclusion, our factor analysis demonstrated that the most common core exacerbating factor of PPPD was the visual factor (sensitivity to visual stimulation), followed by the active-motion factor (sensitivity to walking and active motion) and the passive-motion/standing factor (sensitivity to passive motion and upright position), respectively. Our cluster analysis demonstrated that PPPD can be divided into three subtypes, namely the visual-dominant subtype, the active motion-dominant subtype, and the mixed subtype. Conventional vestibular tests failed to identify the characteristics of each subtype.

## Data Availability Statement

The raw data supporting the conclusions of this article will be made available by the authors, without undue reservation.

## Ethics Statement

The studies involving human participants were reviewed and approved by the Institutional Review Board of Niigata University Medical and Dental Hospital. The patients/participants provided their written informed consent to participate in this study.

## Author Contributions

CY and AH contributed to the conception and design of the manuscript. YM, MK, TY, SO, SI, and KT made substantial contributions to the conception of the work and were responsible for data collection. CY wrote the manuscript. All authors contributed to manuscript revision, read, and approved the submitted version.

## Conflict of Interest

The authors declare that the research was conducted in the absence of any commercial or financial relationships that could be construed as a potential conflict of interest.
